# Evaluation of the prognosis of elderly patients with heart failure by Monocyte-to-High-Density Lipoprotein Ratio, Neutrophil Gelatinase-Associated Lipocalin, and Angiotensin II

**DOI:** 10.3389/fcvm.2025.1609798

**Published:** 2025-06-16

**Authors:** Huadong Liu, Feng Gan, Kun Fu, Liyun Liu, Tingting Wang, Hong Yang, Yudong Fan

**Affiliations:** ^1^ Department of Cardiology, Beijing Aerospace General Hospital, Beijing, China; ^2^ Department of Cardiology, Beijing Emergency General Hospital, Beijing, China

**Keywords:** MHR, NGAL, heart failure, prognosis, Ang II

## Abstract

**Objective:**

The objective of this research was to investigate the correlation between the Monocyte-to-high-density lipoprotein-cholesterol ratio (MHR), Neutrophil gelatinase-associated lipocalin (NGAL), and Angiotensin II (Ang II) with both short-term and long-term mortality rates in elderly patients with heart failure (HF).

**Methods:**

A retrospective cohort study was conducted, encompassing elderly HF patients hospitalized from 2020 to 2023. Multivariable logistic regression analysis was employed to assess the relationship between MHR, NGAL, Ang II, and mortality risk.

**Results:**

The predictive power of these biomarkers for mortality in patients with HF was determined using the area under the receiver operating characteristic curve (AUC). Each of the biomarkers—MHR, NGAL, and Ang II—was linked to an increased risk of mortality at one month (OR = 1.007, 95% CI: 1.003–1.012), (OR = 1.004, 95% CI: 1.001–1.007), (OR = 1.002, 95% CI: 1.001–1.004) and at one year (OR = 1.007, 95% CI: 1.002–1.011), (OR = 1.004, 95% CI: 1.001–1.008), (OR = 1.003, 95% CI: 1.001–1.006) in the elderly patients with HF. The AUC for MHR, NGAL, and Ang II in forecasting one-month mortality were 0.740 (95% CI: 0.668–0.811), 0.659 (95% CI: 0.581–0.738), and 0.628 (95% CI: 0.547–0.710), respectively. For one-year mortality, the AUC values were 0.728 (95% CI: 0.655–0.800), 0.641 (95% CI: 0.560–0.721), and 0.627 (95% CI: 0.546–0.708), respectively. The optimal thresholds for MHR, NGAL, and Ang II in predicting one-month mortality were identified as 0.52, 85 ng/ml, and 25 pg/ml, respectively, while for one-year mortality, the thresholds were 0.50, 70 ng/ml, and 24 pg/ml, respectively.

**Conclusions:**

MHR, NGAL, and Ang II emerge as promising indicators for mortality prediction in HF patients. Among these, MHR stands out as potentially the most reliable predictor of mortality in the elderly with HF.

## 1 Introduction

Heart failure (HF), a clinical condition arising from the heart's structural or functional defects, is a chronic and often worsening disease (1). It significantly contributes to the global burden of cardiovascular disease, leading to considerable morbidity and mortality (2–4). In China, HF is a major public health concern with a prevalence of approximately 1.3% in the general population. The mortality rate for HF patients in China is high, with a reported one-year mortality rate of around 30% and a five-year mortality rate reaching 50%. Additionally, the rehospitalization rate within six months of discharge is as high as 40%, further highlighting the severity and complexity of managing this condition. Despite a decade of advancements in pharmacological interventions and medical devices, the survival outlook for HF patients has not significantly improved (5). The condition is widespread, affecting millions, with a 1-year mortality rate of 30% and a 5-year rate that reaches 75% (6). This underscores the critical need for effective prognostic assessments to guide patient care.

The Monocyte-to-High-Density Lipoprotein Cholesterol Ratio (MHR) is a relatively new biomarker that provides insight into the balance between monocyte inflammation and the protective role of High-Density Lipoprotein Cholesterol (HDL-C) (7). It has shown potential in predicting clinical outcomes, possibly with greater accuracy than individual measures of monocytes or HDL-C. MHR's predictive power for patient outcomes has been suggested across various diseases (8–11). Research has indicated that an elevated MHR is associated with a higher risk of cardiovascular mortality (8–11). However, its role as an independent predictor of mortality in HF, particularly in older adults, is still under investigation.

Neutrophil Gelatinase-Associated Lipocalin (NGAL), a protein released by neutrophils (12), has been linked to the decline in kidney function and the risk of mortality due to renal complications (13–15). It also plays a role in diagnosing cardiovascular diseases (16, 17). Studies have shown that higher NGAL levels in the blood are associated with an increased risk of mortality in HF patients (18). However, the relationship between NGAL and the prognosis of HF in the elderly has not been extensively studied. The renin-angiotensin system (RAS), when not functioning properly, is a key factor in HF and contributes to heart muscle changes (19, 20). Angiotensin II (Ang II) is a central component of the Renin-Angiotensin System (RAS) and is associated with numerous cardiovascular issues (21–23). However, the connection between Ang II levels and outcomes in older HF patients has not been well-studied.

This study aims to explore the association between MHR, NGAL, and Ang II with the risk of mortality over both short and long-term periods in HF patients, with a focus on their potential as prognostic indicators in clinical practice.

## 2 Methods

### 2.1 Study design and patients

A retrospective analysis of patients with HF data was conducted using electronic medical records at Department of Cardiology, First Affiliated Hospital of Chongqing Medical University, between 2020 and 2023. Patients with HF was diagnosed by a consensus of multiple experienced physicians, taking into account patient history (A history of chronic heart failure, including prior episodes of decompensation, hospitalizations for HF, or a history of chronic dyspnea and fatigue), symptoms (Presence of typical symptoms of HF, such as dyspnea on exertion, paroxysmal nocturnal dyspnea, orthopnea, and peripheral edema.), B-type Natriuretic Peptide (BNP) levels (Elevated BNP levels were considered indicative of HF, with a cutoff value of ≥35 pg/ml for BNP and ≥125 pg/ml for NT-proBNP), and echocardiography findings [Echocardiography was performed to assess the left ventricular ejection fraction (LVEF). Patients with an LVEF of ≤40% were classified as having heart failure with reduced ejection fraction (HFrEF), while those with an LVEF of >40% were further evaluated for other signs of HF, such as left ventricular dilation or diastolic dysfunction.). We have specified that the diagnosis of HF patients followed the 2016 ESC Guidelines for the Diagnosis and Treatment of Acute and Chronic Heart Failure (24).

Additional patient data collected included the New York Heart Association (NYHA) functional class, estimated glomerular filtration rate (eGFR), and history of renal failure. The eGFR was calculated using the Chronic Kidney Disease Epidemiology Collaboration (CKD-EPI) formula. The history of renal failure was defined as a history of chronic kidney disease (CKD) with an eGFR <60 ml/min/1.73 m^2^ or a history of acute kidney injury (AKI).

Mortality data were obtained through a combination of electronic health records and telephone follow-up with patients or their families. The primary outcome was one-year mortality, with one-month mortality as a secondary endpoint. Mortality was recorded as any death occurring within 1-month or 1-year post-admission. In addition to MHR, NGAL, and Ang II, we also analyzed the association of the Lymphocyte-to-HDL Ratio (LHR) with mortality risk. Patients were included if they were 65 years or older, fulfilled HF diagnostic criteria, and had all necessary clinical data. Exclusions were applied to those with current or chronic infections, advanced liver or kidney disease (defined as Child-Pugh class C), advanced kidney disease (defined as eGFR <30 ml/min/1.73 m^2^), blood disorders or cancer, congenital heart conditions, autoimmune diseases, or who discontinued therapy. Specifically, patients with liver cirrhosis and significant kidney disease were excluded to avoid potential confounding effects on Angiotensin II (Ang II) levels. The study cohort comprised 225 HF patients who satisfied the inclusion and exclusion criteria. Ethical approval was secured from the Institutional Review Board of the First Affiliated Hospital of Chongqing Medical University, with patient consent waived for this retrospective analysis.

### 2.2 Outcomes

Our primary outcome was one-year mortality, with one-month mortality as a secondary endpoint. Mortality was recorded as any death occurring within 1-month or 1-year post-admission.

### 2.3 Data collection

Demographic details such as gender, Body Mass Index (BMI), and comorbid conditions were collected from electronic health records. BMI was calculated using the standard formula: BMI = weight (kg)/height^2^ (m^2^). Initial admission blood tests assessed High-Density Lipoprotein Cholesterol (HDL-C), Low-Density Lipoprotein Cholesterol (LDL-C), C-Reactive Protein (CRP), White Blood Cell count (WBC), monocyte count, and Left Ventricular Ejection Fraction (LVEF). HDL-C and LDL-C levels were measured using an enzymatic colorimetric method with a fully automated biochemical analyzer (Cobas 8000, Roche Diagnostics, Switzerland). CRP levels were determined using a high-sensitivity immuno-turbidimetric assay (Cobas 8000, Roche Diagnostics, Switzerland). WBC and monocyte counts were obtained from a complete blood count (CBC) using a hematology analyzer (Sysmex XN-9000, Sysmex Corporation, Japan).

Additionally, details of prescribed medications, including beta-blockers (e.g., bisoprolol, metoprolol), ACE inhibitors (e.g., lisinopril, enalapril), ARBs (e.g., losartan, valsartan), ARNI (e.g., sacubitril/valsartan), digoxin, and SGLT2 inhibitors (e.g., dapagliflozin, empagliflozin) were collected. MHR was calculated by dividing the monocyte count by HDL-C levels and was expressed in units of 10^9^/mmol. LHR was calculated by dividing the lymphocyte count by HDL-C levels and was expressed in units of 10^9^/mmol.

NGAL levels were quantified using the Triage® NGAL Test from Biosite Inc.(San Diego, CA, USA) from serum samples.NGAL levels were quantified using the Triage® NGAL Test from Biosite Inc. (San Diego, CA, USA) from serum samples. The assay is a sandwich immunoassay with a detection limit of 10 ng/ml. The intra-assay coefficient of variation (CV) was less than 10%, and the inter-assay CV was less than 12%. Measurements were performed in a blinded manner with respect to clinical outcomes. Angiotensin II levels were measured using a commercially available ELISA kit (Cat. No. EK0514, Boster Biological Technology, Pleasanton, CA, USA) from plasma samples. The assay has a detection limit of 1 pg/ml. The intra-assay CV was less than 8%, and the inter-assay CV was less than 10%. Measurements were performed in a blinded manner with respect to clinical outcomes.

### 2.4 Statistical analysis

Continuous variables are reported as mean ± SD or median [M (Q1, Q3)], with group comparisons made using *t*-tests or Wilcoxon rank sum tests. Categorical variables are presented as counts and percentages [*n* (%)], with group comparisons conducted via chi-square tests. Univariate analysis was used to detect potential HF risk factors. Cox proportional hazards regression analysis was employed to evaluate the impact of MHR, NGAL, Ang II, and LHR on mortality risk (1-month and 1-year) among HF patients. Results are presented as HRs with 95% CIs. The assumption of proportional hazards was tested and confirmed for all models. The predictive power of MHR, NGAL, Ang II and LHR for mortality in patients with HF was assessed using the ROC curve's AUC. All *p*-values reported are two-sided, with *P* < 0.05 indicating statistical significance.

To account for potential confounders, the statistical model was adjusted for variables such as age, sex, atrial fibrillation, monocytes, HDL-C, LVEF, and the presence of comorbid conditions like hypertension, diabetes mellitus, and dyslipidemia. Additionally, renal function (estimated glomerular filtration rate, eGFR) was included in the model to address concerns related to kidney disease. Medication adherence was assessed through medical records and patient interviews, and adherence rates were included as covariates in the analysis.

Given the relatively small sample size (*n* = 225) and low event rates (15.6% one-year mortality), we performed a *post-hoc* power calculation to assess the statistical power of our study. The power calculation was based on the primary outcome of one-year mortality, with an assumed effect size of 1.5 for the hazard ratios of the biomarkers. The power analysis indicated that our study had 80% power to detect a significant difference at a two-sided alpha level of 0.05. Sensitivity analyses were conducted to assess the robustness of our findings, including analyses stratified by age, sex, and presence of comorbidities. The results were consistent across these strata, confirming the stability of our findings.

For handling missing data, we used multiple imputation techniques to impute missing values for key variables. The imputation model included all variables used in the primary analysis, ensuring that the imputed values were consistent with the observed data. Variables with skewed distributions (e.g., NGAL, Ang II) were log-transformed to improve the normality of the data, which is essential for the validity of the statistical tests used. Statistical processing was done with SPSS Statistics 26.0 and GraphPad Prism 8.

## 3 Results

### 3.1 Characteristics of patients

After the exclusion of 31 individuals, a total of 225 patients with HF were incorporated into the analysis. A summary of the characteristics for both the deceased and surviving patients with HF is outlined in Table 1. The cohort included 190 survivors and 35 non-survivors. Within this group, 25 individuals (11.1%) succumbed within the first month, and the one-year mortality rate was 15.6%. Compared with survivors group, non-survivors group were male (68.6% vs. 62.1%, *P* = 0.047), had a prior history of atrial fibrillation (11.4% vs. 4.2%, *P* = 0.024), ARB use (8.6% vs. 28.4%, *P* = 0.015), ARNI use (37.1% vs. 62.1%, *P* = 0.022). Beta-blockers: 110 patients (57.9%) on bisoprolol (mean dose: 5 mg/day), 20 patients (10.5%) on metoprolol (mean dose: 50 mg/day). ACE inhibitors: 40 patients (21.1%) on lisinopril (mean dose: 20 mg/day), 5 patients (2.6%) on enalapril (mean dose: 10 mg/day). ARBs: 54 patients (28.4%) on losartan (mean dose: 50 mg/day), 3 patients (1.6%) on valsartan (mean dose: 80 mg/day). ARNI: 118 patients (62.1%) on sacubitril/valsartan (mean dose: 200 mg/day). Digoxin: 94 patients (49.5%) on digoxin (mean dose: 0.125 mg/day). SGLT2 inhibitors: 25 patients (13.2%) on dapagliflozin (mean dose: 10 mg/day), 6 patients (3.2%) on empagliflozin (mean dose: 10 mg/day). As for laboratory parameters, Monocytes (0.55 (0.43–0.67) vs. 0.51 (0.41–0.63), *P* = 0.033), HDL-C (1.03 (0.89–1.22) vs. 1.09 (0.93–1.29), *P* = 0.028), NGAL (125 (57–274) vs. 62 (46–146), *P* = 0.018), Ang-II (27.6 ± 4.5 vs. 23.5 ± 3.8, *P* = 0.023) and MHR (0.58 (0.38–0.79) vs. 0.45 (0.35–0.56), *P* = 0.004) reached statistical significance. However, no significant intergroup differences can be observed regarding WBC, CRP, LDL-C, and history of hypertension, dyslipidemia, and diabetes mellitus between survival and death group. It is worth noting that the LVEF was significantly lower in the non-survivors group compared to the survivors group (41.8 ± 8.8 vs. 51.4 ± 4.6, *P* = 0.019), which may have an impact on the analysis of mortality.

**Table 1:** Characteristics of patients who did and did not survive the 1-year mark.

Characteristics	Survival *n* = 190	Death *n* = 35	*P*
Age	75.9 ± 6.8	76.4 ± 7.5	0.372
Male	118 (62.1%)	24 (68.6%)	0.047
BMI	23.9 ± 4.4	23.5 ± 3.9	0.269
Hypertension	103 (54.2%)	22 (62.9%)	0.069
Atrial fibrillation	8 (4.2%)	4 (11.4%)	0.024
Diabetes mellitus	37 (19.5%)	9 (25.7%)	0.077
Dyslipidemia	97 (51.1%)	17 (48.6%)	0.077
Monocytes 10^9^/L	0.51 (0.41–0.63)	0.55 (0.43–0.67)	0.033
WBC, 109/L	7.21 (5.91–8.41)	7.61 (6.43–8.71)	0.071
HDL-C (mmol/L)	1.09 (0.93–1.29)	1.03 (0.89–1.22)	0.028
LDL-C (mmol/L)	2.67 (2.10–3.24)	2.55 (1.92–3.26)	0.290
CRP (mg/L)	17.2 ± 12.3	18.8 ± 12.4	0.642
LVEF (%)	51.4 ± 4.6	41.8 ± 8.8	0.019
Beta-blocker	110 (57.9%)	20 (57.1%)	0.235
ACEI	40 (21.1%)	5 (14.3%)	0.067
ARB	54 (28.4%)	3 (8.6%)	0.015
ARNI	118 (62.1%)	13 (37.1%)	0.022
Digoxin	94 (49.5%)	15 (42.9%)	0.072
Dapagliflozin	25 (13.2%)	6 (17.1%)	0.227
MHR	0.45 (0.35–0.56)	0.58 (0.38–0.79)	0.004
NGAL	62 (46–146)	125 (57–274)	0.018
Ang-II	23.5 ± 3.8	27.6 ± 4.5	0.023

### 3.2 Association between MHR, NGAL and Ang Ⅱ mortality

Table 2 shows the association of MHR, NGAL, and Ang II with mortality in elderly patients with heart failure. MHR (HR = 1.086, 95% CI: 1.044–1.131, *P* < 0.001), NGAL (HR = 1.006, 95% CI: 1.004–1.009, *P* < 0.001), and Ang-II (HR = 1.005, 95% CI: 1.003–1.008, *P* < 0.001) were associated with an increased risk of 1-month mortality. Similar trends were observed for 1-year mortality (*P* < 0.05). After adjustment, MHR (HR = 1.007, 95% CI: 1.003–1.012, *P* < 0.001), NGAL (HR = 1.004, 95% CI: 1.001–1.007, *P* = 0.017), Ang II (HR = 1.002, 95% CI: 1.001–1.004, *P* = 0.015), and LHR (HR = 1.002, 95% CI: 1.001–1.003, *P* = 0.023) were still associated with an increased risk of 1-month mortality and 1-year mortality in elderly patients with HF.

**Table 2:** The association of MHR, NGAL and Ang Ⅱ with mortality in elderly patients with heart failure.

Variables	Univariable model		Multivariable model	
	OR (95% CI)	*P*	OR (95% CI)	*P*
1-month mortality				
MHR	1.086 (1.044–1.131)	<0.001	1.007 (1.003–1.012)	<0.001
NGAL	1.006 (1.004–1.009)	<0.001	1.004 (1.001–1.007)	0.017
Ang-II	1.005 (1.003–1.008)	<0.001	1.002 (1.001–1.004)	0.015
1-year mortality				
MHR	1.097 (1.055–1.140)	<0.001	1.007 (1.002–1.011)	0.002
NGAL	1.007 (1.004–1.011)	<0.001	1.004 (1.001–1.008)	0.023
Ang-II	1.006 (1.003–1.009)	<0.001	1.003 (1.001–1.006)	0.025

Multivariable model of MHR, NGAL and Ang Ⅱ adjusted for potential indicators such as sex, atrial fibrillation, monocytes, HDL-C et al. OR, odds ratio; CI, confidence interval.

### 3.3 Predictive ability of MHR, NGAL and Ang Ⅱ for mortality in patients with heart failure

**Figure 1:**
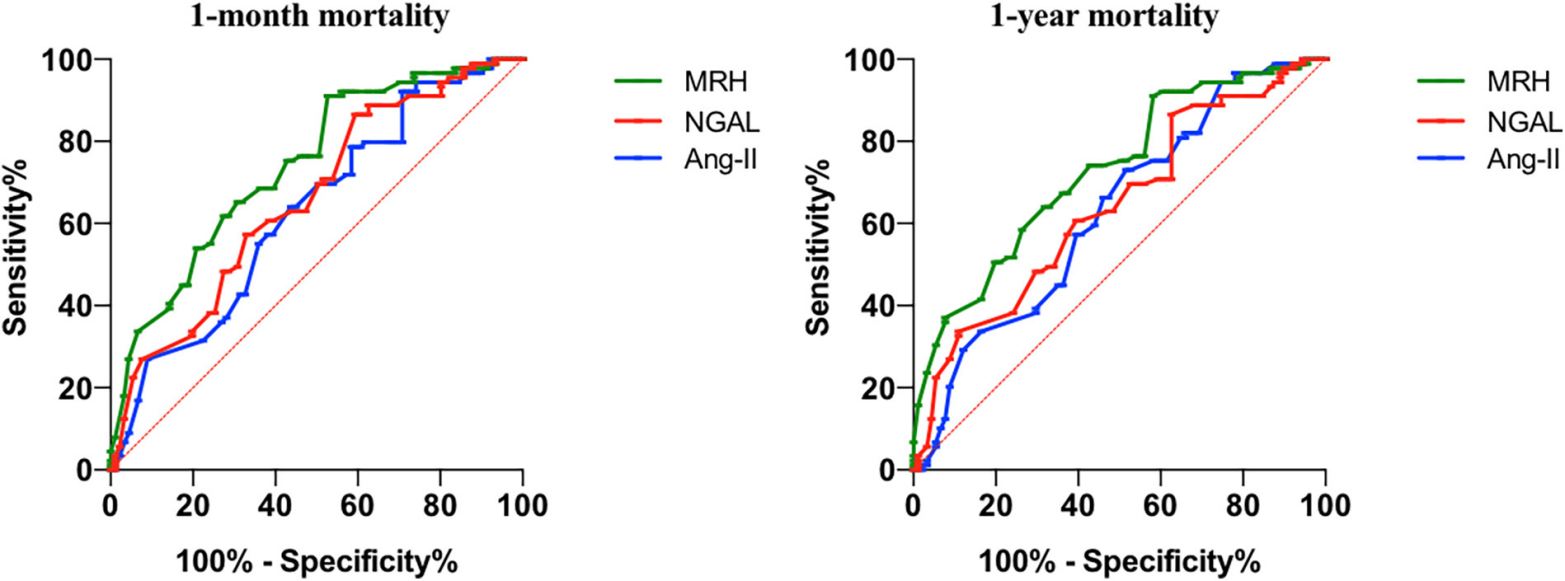
The ROC curve of the MHR, NGAL and Ang Ⅱ for predicting 1-month mortality and 1-year mortality in patients with heart failure.

Table 3 presents the AUC values of MHR, NGAL, and Ang II in predicting 1-month mortality and 1-year mortality in elderly patients with heart failure. The AUC values of MHR, NGAL, and Ang II in predicting 1-month mortality were 0.740 (95% CI: 0.668–0.811), 0.659 (95% CI: 0.581–0.738), and 0.628 (95% CI: 0.547–0.710), respectively. For 1-year mortality, the AUC values were 0.728 (95% CI: 0.655–0.800), 0.641 (95% CI: 0.560–0.721), and 0.627 (95% CI: 0.546–0.708), respectively. The differences in AUC values between MHR and the other biomarkers were statistically significant (*P* < 0.05), indicating that MHR had a slightly higher predictive accuracy for both 1-month and 1-year mortality compared to NGAL and Ang II.The optimal thresholds for MHR, NGAL, and Ang II in predicting 1-month mortality were identified as 0.52, 85 ng/ml, and 25 pg/ml, respectively, while for 1-year mortality, the thresholds were 0.50, 70 ng/ml, and 24 pg/ml, respectively. These thresholds were determined using the Youden index, which maximizes the sum of sensitivity and specificity for each biomarker. MHR had a slightly higher AUC values than AngⅡ and NGAL for predicting 1-month mortality (0.740 vs. 0.659 vs. 0.628) and 1-year mortality (0.728 vs. 0.641 vs. 0.627). The ROC curve of the MHR, NGAL and Ang Ⅱ for predicting 1-month mortality and 1-year mortality in patients with heart failure was shown in Figure 1.

**Table 3:** Predictive ability of MHR, NGAL and Ang Ⅱ for mortality in patients with heart failure.

Outcomes	Variable	AUC (95% CI)	Optimal threshold	Sensitivity	Specificity
1-month mortality	MHR	0.740 (0.668–0.811)	0.52	70%	75%
	NGAL	0.659 (0.581–0.738)	85 ng/ml	65%	60%
	Ang-II	0.628 (0.547–0.710)	25 pg/ml	60%	55%
1-year mortality	MHR	0.728 (0.655–0.800)	0.50	68%	72%
	NGAL	0.641 (0.560–0.721)	70 ng/ml	62%	58%
	Ang-II	0.627 (0.546–0.708)	24 pg/ml	58%	53%

AUC, the area under the receiver operating characteristic curve.

## 4 Discussion

This study investigated the link between MHR, NGAL and Ang Ⅱ with mortality rates in senior individuals suffering from HF. Our findings indicate that elevated levels of MHR, NGAL, and Ang II are correlated with a heightened risk of both 1-month and 1-year mortality among the elderly HF population. These biomarkers demonstrate potential as predictors of both immediate and extended survival outcomes in HF patients. The addition of LHR to our analysis provides further insight into the inflammatory and immune status of patients with HF, which may contribute to a more comprehensive understanding of the disease prognosis. These biomarkers demonstrate potential as predictors of both immediate and extended survival outcomes in HF patients.

Heart failure is characterized by the heart's diminished ability to pump blood effectively due to structural or functional irregularities. The condition is marked by increased hemodynamic load, ischemia-induced dysfunction, and ventricular remodeling (1, 25, 26). Systemic inflammation significantly contributes to the progression of HF, influencing the release of pro-inflammatory cytokines, activation of immune responses, and endothelial inflammation (27). MHR, a marker reflecting the interplay between monocyte inflammation and HDL-C's anti-inflammatory properties, has been implicated in cardiovascular events (28, 29). It may serve as a valuable tool for risk stratification and prognosis assessment in HF. Prior research has identified high MHR as a predictor of cardiovascular events in chronic kidney disease (29) and associated with disease severity in coronary artery disease (30, 31). Our study reveals that HF patients with a high MHR are at an increased risk of mortality, with MHR showing a marginally better predictive capacity than NGAL and Ang II. MHR's routine clinical measurement suggests its practicality for widespread use. It exhibited slightly superior AUC values for 1-month and 1-year mortality prediction compared to NGAL and Ang II, suggesting its robustness as a prognostic indicator. Clinically, MHR could assist in identifying high-risk patients and monitoring disease progression and inflammation levels. Further research should explore whether interventions targeting MHR, such as anti-inflammatory therapies, could enhance patient outcomes. Monitoring MHR could be pivotal for risk identification and survival improvement, presenting as a cost-effective risk assessment tool.

Potassium-sparing diuretics: Although potassium-sparing diuretics like spironolactone are commonly prescribed to patients with heart failure, their use was not the primary focus of this study. However, we did collect data on the prevalence of spironolactone use, with 39.5% of patients receiving this medication. Future studies should further investigate the impact of potassium-sparing diuretics on mortality and other clinical outcomes in heart failure patients, especially in the context of other medications and comorbidities.

NGAL has been recognized for its mortality-predicting potential in HF patients, irrespective of chronic kidney disease status (18). Our study affirms these findings, establishing NGAL's association with 1-month and 1-year mortality risks in elderly HF patients. However, the necessity for specialized kits to measure NGAL suggests a need for more accessible prognostic indicators in HF. This research compared the predictive power of MHR, NGAL, and Ang II for mortality in HF, contributing to the understanding of their roles in patient survival. Nonetheless, the study has some limitations. As a single-center investigation, it is susceptible to selection bias, and future multi-center studies are warranted to substantiate these findings. Additionally, the reliance on single-timepoint measurements for MHR, NGAL, and Ang II may not capture the full dynamic range of these biomarkers.

However, there are several limitations to this study. First, as a single-center investigation, it is susceptible to selection bias, and future multi-center studies are warranted to substantiate these findings. Second, the reliance on single-timepoint measurements for MHR, NGAL, and Ang II may not capture the full dynamic range of these biomarkers. Third, mortality data were obtained through a combination of electronic health records and telephone follow-up. While efforts were made to verify the cause of death, it is possible that some patients may have died from causes unrelated to HF, such as accidents or other non-cardiovascular events. This potential bias should be considered when interpreting the results. Additionally, the lower LVEF in the non-survivors group compared to the survivors group may have influenced the analysis of mortality. Future studies should explore the impact of LVEF on mortality in more detail to better understand its role as a potential confounder.

The clinical implications of our findings are significant. MHR, NGAL, and Ang II could potentially be incorporated into clinical practice as part of a comprehensive risk stratification strategy for elderly HF patients. These biomarkers could help identify high-risk patients who may benefit from more intensive monitoring and targeted interventions. For instance, elevated MHR levels could prompt clinicians to consider anti-inflammatory therapies or more frequent follow-up visits. Similarly, elevated NGAL and Ang II levels could signal the need for closer monitoring of renal function and cardiovascular status.

Incorporating these biomarkers into existing risk stratification models, such as the MAGGIC and Seattle HF scores, could enhance their predictive accuracy and clinical utility. Future research should focus on validating the use of these biomarkers in combination with established models to improve risk prediction and guide clinical decision-making. Additionally, interventions aimed at modulating MHR, such as anti-inflammatory therapies, could be explored in clinical trials to determine their impact on patient outcomes.

In summary, MHR, NGAL, and Ang II are linked to increased short-term and long-term mortality risks in elderly HF patients, with MHR showing a modest edge in predictive accuracy over the others. MHR emerges as a promising biomarker for mortality prediction in HF, warranting further investigation into its trajectory and impact on patient survival.

## Data Availability

The original contributions presented in the study are included in the article/Supplementary Material, further inquiries can be directed to the corresponding author.
